# Elevated BCRP Transporter and Altered NF‐кB Pathway Mediate Zoledronic Acid Resistance in MCF‐7 Cells

**DOI:** 10.1002/jbt.70397

**Published:** 2025-07-14

**Authors:** Öykü Irmak Dikkatli, Yunus Emre Cavlak, Yaprak Dönmez Çakıl, Sueda Atılkan, Erkan Yurtcu, Özlem Darcansoy İşeri

**Affiliations:** ^1^ Department of Molecular Biology and Genetics, Institute of Science Başkent University Ankara Türkiye; ^2^ TENMAK, Boron Research Institute Ankara Türkiye; ^3^ Department of Molecular Biology and Genetics, Faculty of Science and Letters Başkent University Ankara Türkiye; ^4^ Department of Medical Biology and Genetics, Faculty of Medicine Maltepe University İstanbul Türkiye; ^5^ Department of Medical Biology, Faculty of Medicine Kırıkkale University Kırıkkale Türkiye

**Keywords:** ABCG2 transporter, EMT, multidrug resistance, NF‐κB pathway, Zoledronic acid

## Abstract

Zoledronic acid (ZA), a bisphosphonate derivate, became the standard for preserving bone structure in cancer. Using various intracellular signaling pathways, including NF‐κB, ZA inhibits tumor cell proliferation, induces apoptosis, and has additive and synergistic effects with cytotoxic agents. However, it has been observed that resistance has developed against ZA. This study aims to explore the underlying mechanisms of ZA resistance in MCF‐7 breast cancer cells by investigating the activity and localization of the human breast cancer resistance protein (BCRP), changes in the NF‐κB pathway, and the markers of epithelial‐mesenchymal transition (EMT). Previously, MCF‐7 cells were stepwise selected in increasing concentrations of ZA and became resistant to 8 µM ZA (MCF‐7/Zol). We determined that BCRP levels were elevated with altered intracellular localization in ZA resistant MCF‐7 cells, and BCRP pump caused a decrease in the substrate accumulation in the MCF‐7/Zol cells whereas no change in intercellular substrate accumulation was observed in parental cells. MCF‐7/Zol cells have increased amount of phosphorylated IκB which is associated with increased nuclear translocation of NF‐κB. Concordantly, BCRP upregulation may be associated with increased nuclear NF‐κB in ZA resistant cells. MCF‐7/Zol cells did not harbor EMT markers. Elucidation of molecular mechanisms of resistance developed against chemotherapeutic agents is important to target critical pathways and proteins to eliminate the resistant clones as well as for determining biomarkers for MDR.

## Graphical abstract

BCRP levels are elevated with altered intracellular localization in ZA resistant MCF‐7 cells, BCRP pump causes a decrease in the specific substrate accumulation in the ZA resistant cells, MCF‐7/Zol cells have increased amount of phosphorylated IκB which is associated with increased nuclear translocation of NF‐κB

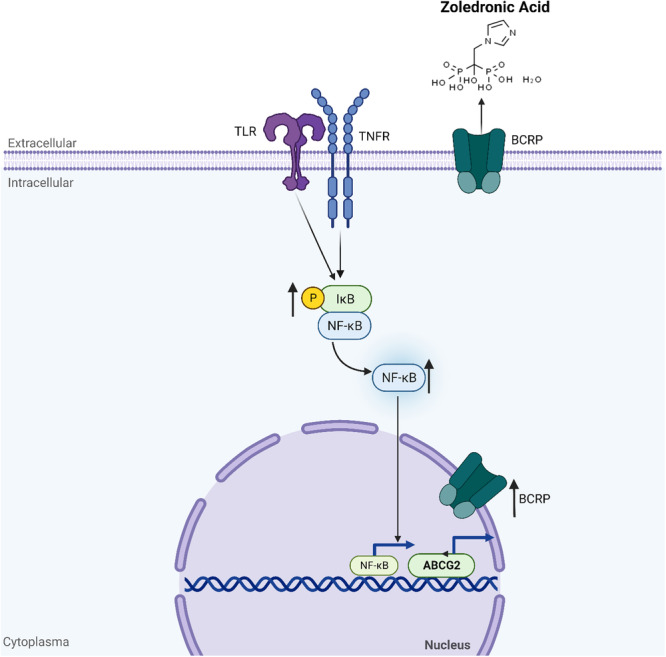

## 1 Introduction

Bone loss is a common consequence of cancer. Patients with solid tumors frequently suffer from decreased bone mineral density and impaired bone health induced by systemic treatments as well as bone metastasis. The latter is particularly a major issue in patients with breast, prostate, and lung cancer and causes substantial morbidity and mortality [1]. Bone metastasis‐related complications including a range of skeletal‐related events (SREs) such as hypercalcemia, spinal cord compression, and pathological fractures are estimated to affect half to two‐thirds of patients with breast cancer [2], highlighting the requirement of therapeutic agents for maintaining bone integrity. Bone metastasis is characterized by the infiltration of bone with circulating cancer cells and includes various cytokines, growth factors, and other molecules, which activate different pathways leading to bone resorption [3].

Reducing bone resorption through the inhibition of osteoclast generation and activity is an established therapeutic strategy to reduce the risk of SREs. Zoledronic acid (ZA), a potent bisphosphonate derivate, became the standard for preserving bone structure for over a decade [4]. A recent meta‐analysis reported a lower incidence of SREs and reduced pain levels following systemic ZA administration in patients with bone metastases [5]. The mechanisms of ZA in the reduction of osteoclast differentiation, survival, and bone resorptive activity have been shown to be associated with various signaling pathways including the noncanonical Wnt signaling through Ca^2+^/calmodulin‐dependent protein kinase II and receptor activator of nuclear factor (NF)‑κB ligand (RANKL)/RANK pathway with its downstream phosphatidylinositol 3‐kinase (PI3K)/protein kinase B (Akt)/mTOR and NF‐κB pathways [3, 6]. Additionally, clinical evidence demonstrated improved long‐time survival rates in patients with cancer regardless of the presence of bone metastasis, thereby indicating the antitumor effects of ZA. Various in vitro and in vivo studies have reported that ZA inhibits tumor cell proliferation, invasion, and migration as well as angiogenesis. ZA also induces apoptosis and has additive and synergistic effects with cytotoxic agents. Additionally, ZA modulates immune responses in the tumor microenvironment [7].

The ability of cancer cells to survive treatment with a wide range of structurally unrelated cytotoxic compounds is defined as multidrug resistance (MDR). Drug efflux through ATP‐binding cassette (ABC) transporters, epithelial‐mesenchymal transition (EMT), the tumor microenvironment, suppression of apoptosis, and development of stemness are among the multiple potential mechanisms that contribute to MDR [8, 9, 10]. As many factors gradually affect cell survival and/or proliferation pathways [11], recently we have also demonstrated that genomic alterations and genomic instability may change depending on the resistance status [12]. Concordantly, a multitude of cellular characteristics undergo significant alteration during the process of EMT. These changes primarily manifest as modifications in cell‐cell and/or cell‐matrix interactions, as well as motility, and invasiveness. Additionally, EMT has been observed to occur in the context of tumor progression in carcinomas, with alterations associated with EMT frequently serving as prognostic indicators.

The human breast cancer resistance protein (BCRP, ABCG2) is among the prominent ABC transporters responsible for anticancer drug efflux together with P‐glycoprotein (P‐gp, MDR1, ABCB1) and multidrug resistance protein 1 (MRP1, ABCC1). BCRP is regulated by multiple signaling pathways, including NF‐κB, P13K/Akt, and the extracellular signal‐regulated kinases 1 and 2 (ERK1/2). Moreover, some signaling pathways, such as Wnt/β‐catenin, are postulated to be linked to BCRP status in stem cells [13]. Its overexpression increases the intracellular accumulation of diverse chemotherapeutic agents, such as mitoxantrone, topotecan, SN‐38, and doxorubicin, and is associated with poor clinical outcomes in several cancer types [14].

The promising progress in preclinical research prompted the investigation of ZA resistance in a variety of cancer cells [15, 16, 17, 18, 19, 20]. However, the precise mechanisms have not been fully elucidated yet. Previously, our group demonstrated the overexpression of two drug efflux proteins, BCRP and the lung resistance protein (LRP; the major vault protein), as well as the upregulation of the antiapoptotic response in ZA‐resistant MCF‐7 breast cancer cells [15]. This study aims to further explore the underlying mechanisms of ZA resistance in breast cancer cells by investigating (i) BCRP activity and localization, (ii) changes in the NF‐κB pathway, and (iii) EMT.

## 2 Materials and Methods

### 2.1 Cell Lines and Culture Conditions

The parental MCF‐7 cell line (MCF‐7/S), which is a model cell line for human mammary carcinoma was donated by the ŞAP Institute (Türkiye). The MCF‐7/Zol cell line was developed by stepwise selection of resistant clones as previously described [15]. MCF‐7/Zol cells became resistant to 8 µM ZA (zoledronic acid monohydrate, USA), and were 3.7‐fold resistant to ZA in comparison to parental cells. Cells were cultured with RPMI 1640 (Capricorn Scientific, Germany) supplemented with 10% (v/v) heat‐inactivated fetal bovine serum (FBS) (Capricorn Scientific), l‐glutamine, and 1% (v/v) penicillin‐streptomycin (France) in a 95% humidified atmosphere of 5% CO_2_ at 37°C (Japan). MCF‐7/Zol cells were cultured in the presence of 8 µM ZA.

### 2.2 Protein Isolation and Western blot

Confluent 75 cm^2^ flasks of MCF‐7/S and MCF‐7/Zol cells were trypsinized, and pelleted in phosphate‐buffered saline (PBS) (Merck KGaA, Germany). Lysis buffer containing a cocktail of protease inhibitors (1 mM phenylmethylsulphonyl fluoride (PMSF); 2 µg/mL aprotinin; 1 µg/mL pepstatin) (Merck) was added to the pellet. Following 30 min ice incubation, nuclei were pelleted at 3400 rpm for 5 min at 4°C. The supernatant containing cell lysate was collected, and samples were stored at −80°C. For the extraction and fractionation of nuclear proteins, EpiQuik Nuclear Extraction Kit II (USA) was used. Protein concentration was measured by Bradford assay [21]. Total and nuclear protein samples were fractionated electrophoretically by SDS‐PAGE (12%) (BioRad, USA) and transferred to a 0.45 µm nitrocellulose membrane (Merck) in a semi‐dry transfer system (Hoefer, USA). The nonspecific binding sites were blocked with 5% (w/v) nonfat dry milk in Tris‐buffered saline with Tween‐20 (TBST). The membrane was then overnight incubated with anti‐BCRP (ELK Biotechnology, USA) (1:1000), anti‐NF‐κB (Abcam, UK) (1:1000), and anti‐IKB‐α (phospho Ser32/36) (ELK Biotechnology) (1:2000) primary antibodies in optimized working dilutions at 4°C. Glyceraldehyde‐3‐phosphate dehydrogenase (GAPDH) (Cell signaling, USA) (1:1000) antibodies were used as internal loading controls for total proteins, whereas TBP (Elabscience, USA) (1:2000) antibody was used for nuclear samples. Detection was performed by using either peroxidase‐conjugated goat anti‐mouse IgG (SouthernBiotech, USA) or goat anti‐rabbit IgG (Abcam) secondary antibodies (1:5000) followed by the application of EZ‐ECL chemiluminescence (Biological Industries, Israel), and visualized with G: Box (SYNGENE, UK). Immunoblots were performed as duplicates, and densitometric analysis was done using ImageJ Software (https://imagej.net/ij/).

### 2.3 Immunofluorescence Labeling of Cells

Immunofluorescence was performed as previously described [22]. Briefly, 10^5^ cells were cultured on coverslips, fixed in cold pure methanol (chilled at −20°C) followed by permeabilization in PBS containing 0.2% (v/v) Triton X‐100 (Merck) (PBST). Next, the cells were incubated in PBST containing 1% (w/v) bovine serum albumin (BSA) (Merck) for blocking. To determine subcellular BCRP localization, polyclonal anti‐BCRP primary antibody (1:100) (ELK Biotechnology) targeting the intracellular region of the BCRP protein and FITC‐conjugated goat anti‐rabbit IgG (H&L) (1:200) (Abbkine, USA) secondary antibody were used. Nuclei were then counter‐stained with Hoechst 33342 (Thermo Scientific, USA). Analysis was performed with a Zeiss LSM700 (Germany) confocal laser scanning microscope. Cells from different areas were captured and evaluated.

### 2.4 Flow Cytometric Analysis

To determine the BCRP pump activity in cells, the intracellular concentration of the fluorescent dye Pheophorbide a (PhA) (#16072, Cayman Chemical, USA), a substrate‐specific to BCRP, was measured by flow cytometry as described by Robey et al. [23]. MCF‐7/S and MCF‐7/Zol cells were loaded with PhA in the absence or presence of the BCRP inhibitor elacridar [24]. PhA and elacridar (#207613, Santa Cruz Biotechnology, USA) were dissolved in DMSO (Merck) at 1 mg/mL (1.69 mM) and 1 mg/mL (1.77 mM), respectively. Cells were trypsinized and centrifuged at 400 g for 5 min and washed 2 times with PBS. Then, cells (10^6^ cells/mL) were incubated for 30 min at 37°C with 1 µM PhA or 1 µM PhA and 1 µM elacridar in RPMI 1640 medium with 10% FBS. Samples were kept on ice to minimize pump activity and washed with cold medium by centrifugation. Measurements were made with BD Accuri C6+ flow cytometer (BD Biosciences, UK) in a cold environment. At least 20,000 events were collected in the “Cells” gate for each sample. PhA fluorescence data were collected from FL‐3 (excitation: 488 nm and emission: > 670 nm), and median fluorescence values were used to calculate the fold change of PhA accumulation. Analyses were performed in triplicates.

### 2.5 Immunohistochemistry

MCF‐7/S and MCF‐7/Zol cell lines were harvested and resuspended in serum‐free media. The suspension was centrifugated for 5 min at 1000 rpm (Thermo Scientific), and the pellets were fixed in 10% (v/v) alcohol‐formalin for 2 h followed by overnight incubation in fresh fixative. After fixation, samples were centrifuged at 2500 rpm for 15 min at RT, and pellets were hardened in freshly prepared 10% (v/v) alcohol‐formalin solution for an additional 24 h at RT. Fixed samples were embedded into paraffin blocks, and 4‐micron sections were taken by a microtome (Leica SM2000R, Germany). The samples were incubated in primary antibodies anti‐Vimentin (mouse, monoclonal) (Ventana, Roche Diagnostics, Switzerland) and anti‐E‐cadherin (mouse, monoclonal) (Ventana, Roche Diagnostics) for 1 h at 42°C. ‘′Ultraview universial 3,3′‐ Diaminobenzidine (DAB‐detection kit) (Ventana) was used, and slides were counterstained with hematoxylin (Ventana, Roche Diagnostics) and Bluing Reagent (Ventana, Roche Diagnostics), and mounted. Micrographs were obtained with Leica DMi8 manual inverted microscope (Germany).

### 2.6 Quantitative Reverse Transcription Polymerase Chain Reaction (RT‐qPCR)

RNA isolation from MCF‐7/S, MCF‐7/Zol, and U87 cell lines was performed using TRI Reagent® (Sigma‐Aldrich, Germany) according to the manufacturer′s instructions. U87, a human glioblastoma cell line was used as a control for mesenchymal marker genes. Optical density at 260 nm and 280 nm was measured for RNA quantification. Five hundred ng of RNA was reverse‐transcribed using TaKaRa PrimeScript™ 1st Strand cDNA Synthesis Kit (TaKaRa, Japan) with both 100 pmol oligo dT primers (42°C, 1 h) and 100 pmol random 6‐mers (30°C, 10 min).

cDNA was used as template for the expression analysis of *CDH1, Vimentin, SNAIL1, and NF‐kB* genes. *TBP* (*TATA‐binding protein*) was used as the housekeeping gene to normalize the expression levels. qPCR was performed in 20 µL reaction mix which contained 4 µL cDNA, 10 µM from each primer set, and the FastStart Essential DNA Green Master mix (Roche) using Thermo Scientific™ PikoReal™Real‐Time PCR System (Thermo Scientific). Primers were selected from different exons of the genes to prevent the amplification of contaminating DNA. The primer sets (Oligomer Biotechnology, Türkiye) and amplicon sizes are listed in Table 1. Each sample run was performed in triplicates with non‐template controls. The relative changes in gene expression levels were calculated according to 2^−∆∆Ct^ method [26].

**Table 1:** Primer sets for qPCR.

Primer sets	Gene accession number		Sequence	Exon/Intron location	Amplicon size (bp)
*CDH1* [25]	ENSG00000039068	F	5′‐GCCTCCTGAAAAAGAGAGTGGAAG‐3′	Exon 10	131
		R	5′‐TGGCAGTGTCTCTCCAAATCCG‐3′	Exon 11	
*Vimentin* [25]	ENSG00000026025	F	5′‐AGGCAAAGCAGGAGTCCACTGA‐3′	Exon 6	100
		R	5′‐ATCTGGCGTTCCAGGGACTCAT‐3′	Exon 7	
*SNAIL1* [25]	ENSG00000124216	F	5′‐CACTATGCCGCGCTCTTTC‐3′	Exon 1	113
		R	5′‐GGTCGTAGGGCTGCTGGAA‐3′	Exon 2	
*NF‐kB*	ENSG00000077150	F	5′‐GGGTGTCCTGCATGTGACTA‐3′	Exon 7	213
		R	5′‐AGTGATGGCTCCTTCTCCCT‐3′	Exon 8	
*TBP*	ENSG00000112592	F	5′‐ACAACAGCCTGCCACCTTAC‐3′	Exon 2	138
		R	5′‐TTTGGAAGAGCAACAAAGGC‐3’	Exon 3	

### 2.7 Statistical Analyses

All data are expressed as mean ± standard error of the means (SE) or standard deviation (SD). All statistical analyses were performed using SPSS 20.0 Software (SPSS Inc., USA). The mean differences between the sensitive and resistant cells were statistically evaluated by *t*‐test at the *p* < 0.05 level. All statistical analyses were conducted on the basis of raw data.

## 3 Results

### 3.1 Elevated BCRP Levels with Altered Intracellular Localization Is Determined in ZA Resistant MCF‐7 Cells

**Figure 1:**
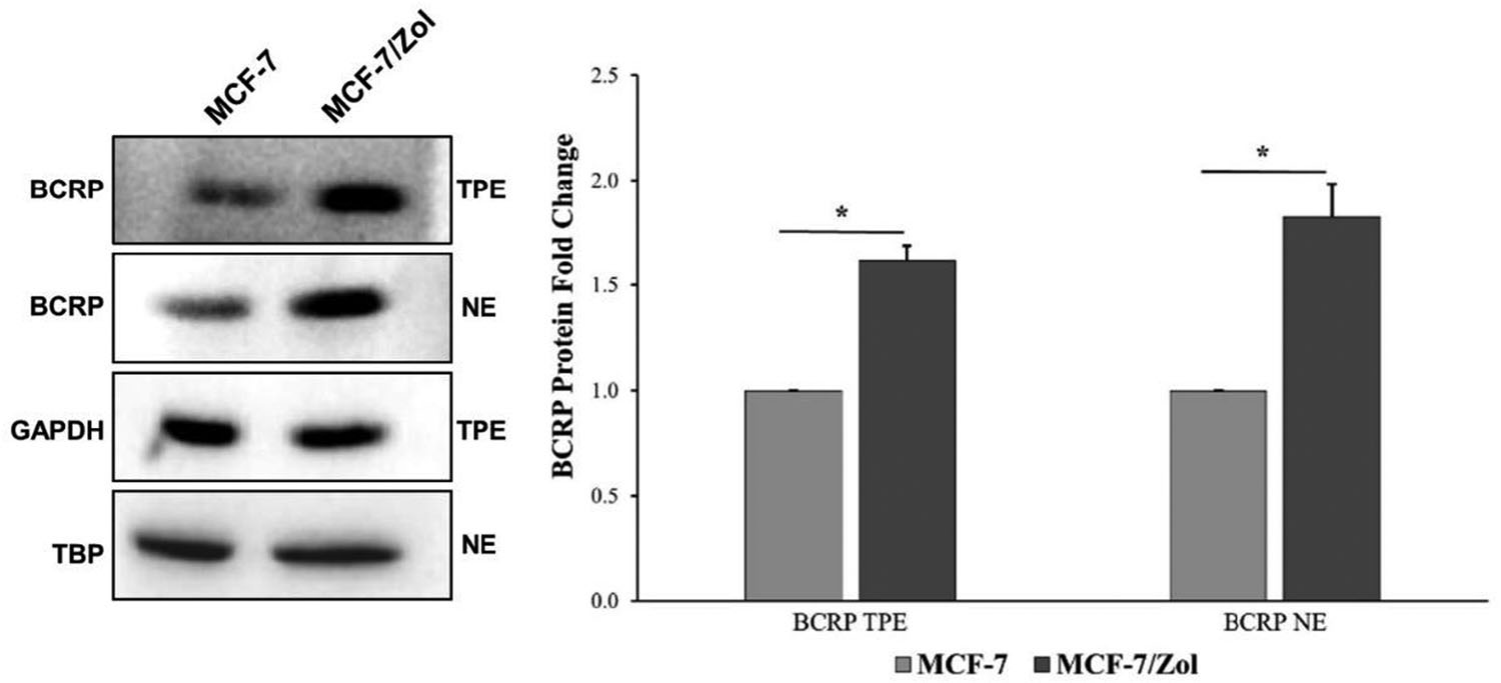
Western blot analysis of BCRP protein; TPE: total protein extract, and NE: nuclear extract. (*) Indicates *p* < 0.05. Error bars represent SE.

According to the western blot results (Figure 1), it was determined that the amount of BCRP in total protein and nuclear lysates increased significantly in MCF‐7/Zol cells compared to that in parental cells.

**Figure 2:**
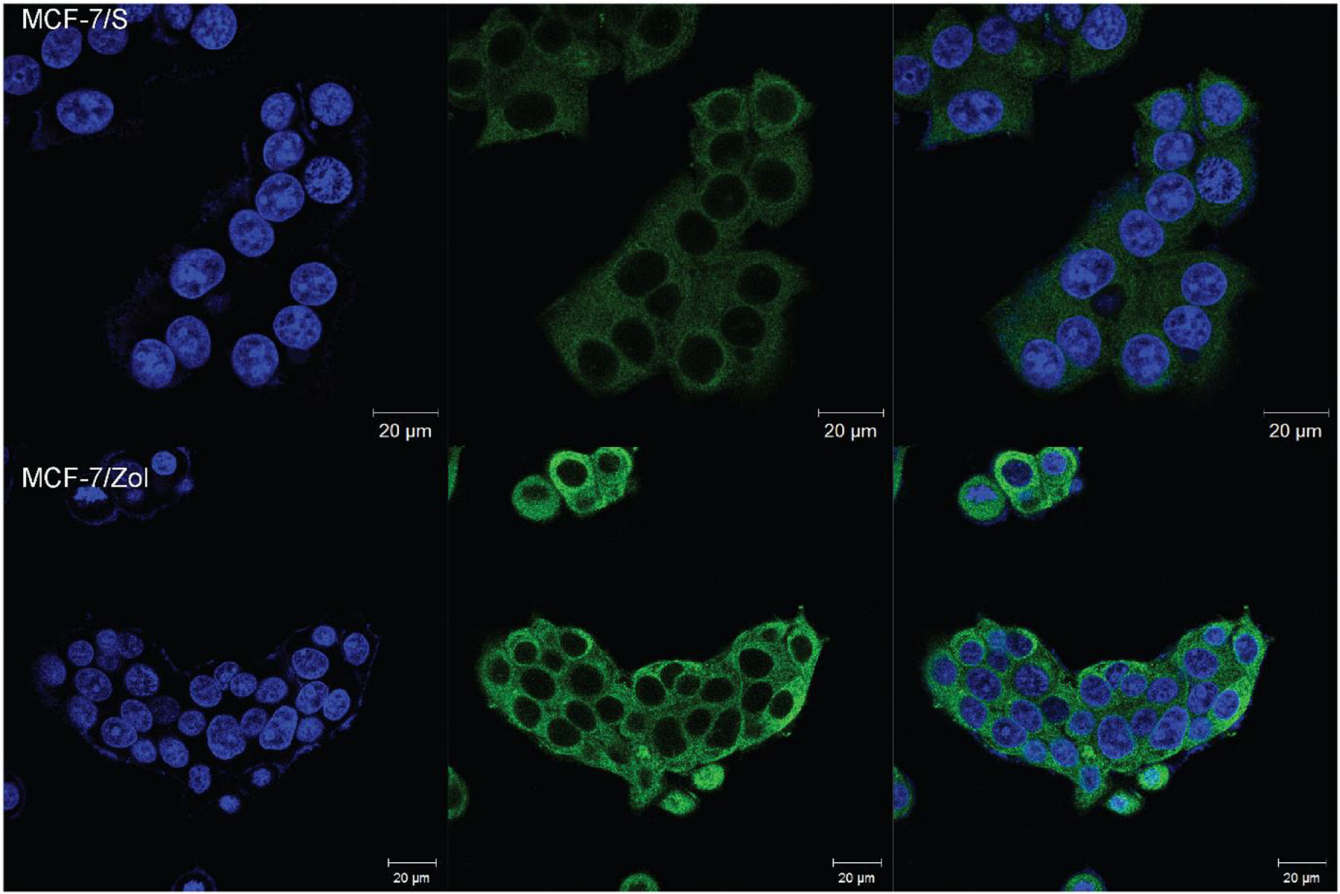
Cellular BCRP localization and distribution were detected by confocal immunofluorescence microscopy in MCF‐7/S (upper panel) and MCF‐7/Zol cells (lower panel). The cells were labeled with anti‐BCRP primary antibody and FITC‐conjugated secondary antibody following fixation and permeabilization. Counterstaining was performed with Hoechst 33342 to observe cell nuclei and analyses were performed with DAPI and GFP filters.

Immunofluorescence analysis was further performed to characterize cellular BCRP localization in MCF‐7 parental and MCF‐7/Zol cells. BCRP was found in the cytoplasm, primarily at the periphery of the nucleus in MCF‐7/S cells. As expected, the signal was weak due to the low endogenous levels of BCRP (Figure 2, upper panel). On the other hand, intense staining corresponding to higher BCRP expression was detected in MCF‐7/Zol cells. BCRP protein was observed both at the plasma membrane and throughout the MCF‐7/Zol cells (Figure 2, lower panel).

### 3.2 BCRP Decreases PHA Accumulation in ZA Resistant Cells

**Figure 3:**
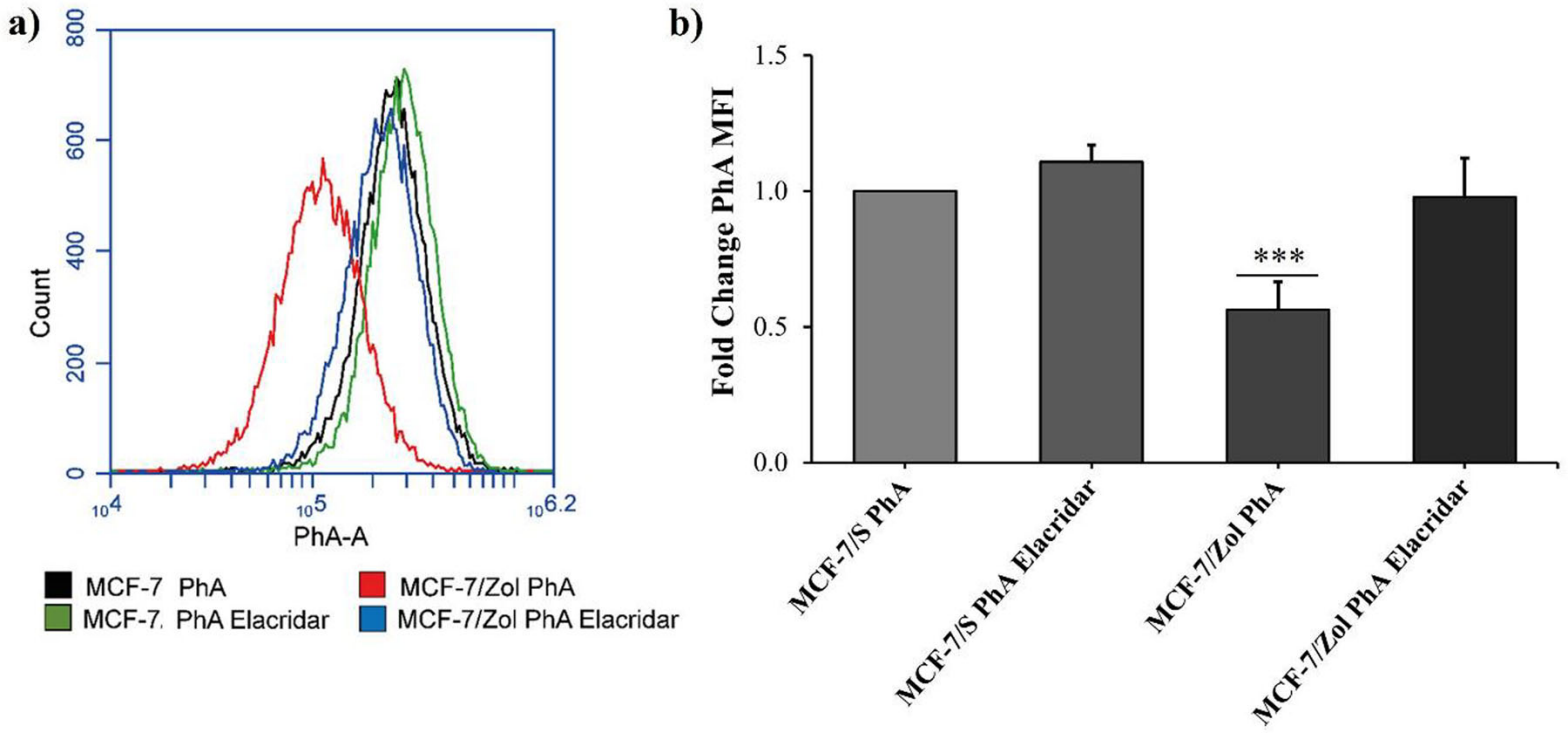
Pheophorbide a (PhA) accumulation in MCF‐7 parental and MCF‐7/Zol cells (a) Histogram demonstrates the accumulation curves in the absence and presence of the potent inhibitor elacridar (b) Median fluorescence values were used to determine the fold changes in PhA accumulation in comparison to that of untreated MCF‐7/S cells. (***) Indicates significance (*p* ≤ 0.0001) between the groups. Error bars represent SE.

To evaluate BCRP‐mediated efflux activity in MCF‐7/Zol cells, the accumulation of BCRP‐specific fluorescent substrate PhA was examined in MCF‐7 parental and MCF‐7/Zol cells in the absence or presence of the potent inhibitor elacridar. The histogram (Figure 3a) demonstrates the PhA accumulation curves with different median fluorescence values, which were used to determine the fold changes in comparison to the untreated parental cells (Figure 3b). Accordingly, whilst MCF‐7/Zol cells accumulated significantly less PhA (0.56 ± 0.10 fold, *p* ≤ 0.0001), the treatment with BCRP inhibitor elacridar restored the PhA fluorescence of MCF‐7/Zol cells to a level similar to that of untreated MCF‐7/S cells (0.98 ± 0.14 fold). Elacridar treatment did not result in a significant change in PhA accumulation in MCF‐7/S cells (1.11 ± 0.06 fold).

### 3.3 NF‐κB Pathway Was Altered in the Za Resistant Cells

**Figure 4:**
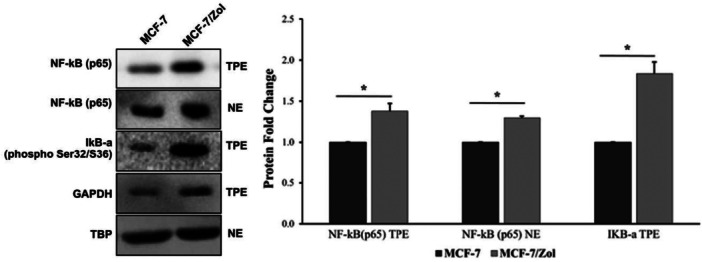
Western blot analysis of genes involved in the NF‐κB pathway; TPE: total protein extract and NE: nuclear extract. (*) Indicates significance (*p* < 0.05) between the groups. Error bars represent SE.

Western blot of the components of the NF‐κB pathway (Figure 4) demonstrated that there is a significant increase in phosphorylated IκB levels in the MCF‐7/Zol cells. A concomitant increase in the cytosolic free NF‐κB levels was also determined in these cells in comparison to the parental cells. Nuclear NF‐κB levels were higher in the MCF‐7/Zol cells, which demonstrates increased nuclear translocation of NF‐κB. In addition to western blot results, NF‐κB mRNA level was determined by RT‐qPCR. 2^−ΔΔCt^ in MCF‐7/S and MCF‐7/Zol cells was calculated as 1.07 and 1.01, respectively, and no significant difference was found.

### 3.4 ZA Resistant Cells Did Not Harbor EMT Markers

**Figure 5:**
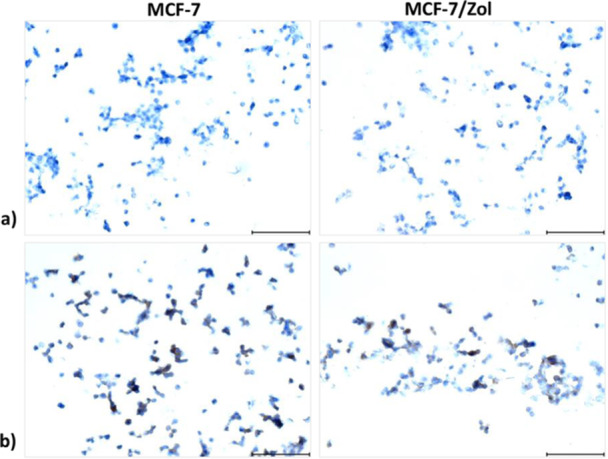
Immunohistochemistry micrographs labeled with (a) vimentin and (b) E‐cadherin antibodies. Scale bars represent 87.1 µm.

According to the immunohistochemistry micrographs (Figure 5), both parental MCF‐7/S and MCF‐7/Zol cells had epithelial E‐cadherin expression, whereas mesenchymal marker vimentin was not observed.

Next, we checked the expression levels of *CDH1* and *VIM* and genes, encoding the E‐cadherin and vimentin, respectively, as well as the EMT regulator transcription factor *SNAIL* gene expression level to confirm immunohistochemistry analysis. Both mesenchymal markers *VIM* and *SNAIL* expression levels were normalized to U87 human glioblastoma cell line, which is known to express mesenchymal markers. According to RT‐qPCR results, *VIM* levels were considerably lower than the U87 cell line, and the expression levels did not differ between MCF‐7/S and MCF‐7/Zol cells (with 2^−ΔΔCt^ of 5.6 × 10^−4^ and 4.2 × 10^−4^, respectively). Similarly, there was not significant difference between the expression levels of *CDH1* (with 2^−ΔΔCt^ 4.2 and 3.87 for MCF‐7/S and MCF‐7/Zol, respectively) and *SNAIL* (with 2^−ΔΔCt^ 1.048 and 1.046 for MCF‐7/S and MCF‐7/Zol, respectively) genes.

## 4 Discussion

In our previous study [15], MCF‐7 cells were selected in increasing concentrations of ZA and became resistant to 8 µM ZA. Considering the concentration of bisphosphonates in plasma has been reported to range from 1 to 3 μM [16], the resistant status of ZA is clinically relevant. MCF‐7/Zol cells have a been shown to exhibit cross‐resistance to paclitaxel, docetaxel, and vincristine, and a synergistic effect of these drugs with ZA has also been demonstrated. Though the action mechanisms of these drugs differ, multidrug resistance phenotype developed in these cells points out at least some common mechanisms to decrease the cytotoxic effects of the drugs. ZA, along with other bisphosphonates, exerts its antitumor effect through apoptosis, and these effects are enhanced with several chemotherapeutics including paclitaxel and doxorubicin [27, 28, 29]. Concordantly, it was demonstrated that the antiapoptotic Bcl‐2 protein increased and the apoptotic Bax protein decreased in the MCF‐7/Zol cells resulting in an increase in the Bcl‐2/Bax ratio.

Several ABC transporter proteins are important components of drug efflux mediated drug resistance developed against structurally and mechanistically unrelated cytotoxic agents. Amongst, the half transporter BCRP protein is one of the least studied. *ABCG2* gene overexpression was shown in MCF‐7/Zol cells, previously. It has been shown that BCRP protein is present in the nuclear membrane of different tumor cell populations [30, 31]. Therefore, determining the subcellular localization of BCRP in cells together with its functionality in intracellular substrate accumulation becomes important in terms of MDR phenotype developed in MCF‐7/Zol cells. In this study, for the determination of the localization and protein levels of BCRP in parental and ZA resistant cells, western blot analysis was performed by using total protein and nuclear extracts together with the immunofluorescence staining. BCRP amount increased in MCF‐7/Zol cells with increased localization at the plasma membrane of cells. Next, we asked the question whether these changes are associated with the increased levels of substrate accumulation in MCF‐7/Zol cells. Accumulation of less PhA, the specific fluorescent substrate, in MCF‐7/Zol cells [23], and restoration of PhA accumulation in the presence of elacridar, a BCRP inhibitor [32], demonstrated presence of functional BCRP transporter proteins in these cells. In another study conducted by our group with the MCF‐7/Zol cells [33], the effect of Biochanin A, an isoflavonoid and BCRP pump inhibitor, on reversal of ZA resistance was examined. MCF‐7/Zol cells treated with Biochanin A became sensitized to ZA in a concentration‐dependent manner, with an approximately 55% reversal of resistance. No other study has been found in the literature that establishes a functional association between the BCRP pump or elevated BCRP levels and ZA resistance.

NF‐κB dimers exist in the cytosol bound to its inhibitor, IκB protein. By the activation of the canonical pathway in cells, the IκB kinase (IKK) complex phosphorylates the IκB protein. Phosphorylated IκB is degraded in the proteasome releasing NF‐κB. NF‐κB (p50/p65) translocates to the nucleus and binds to DNA. The increased amount of phosphorylated IκB in MCF‐7/Zol cells is associated with increased nuclear translocation together as well as increased amount of cytosolic free NF‐κB considering that the NF‐κB mRNA levels remained unchanged. NFκB provides a mechanistic link between inflammation and cancer and is an important factor controlling the ability of both neoplastic and malignant cells to resist apoptosis. Genes known to be important actors in processes such as cell proliferation, immunity, cell survival, metastasis, angiogenesis, and invasion contain regions that bind NF‐κB. NFκB activates genes that are responsible for the suppression of intrinsic or extrinsic apoptotic pathways by upregulating antiapoptotic genes and downregulating p53 levels [34]. Interestingly, Bcl‐2 promoter has also NF‐κB binding sites [35], which can be correlated to our previous findings demonstrating increased Bcl‐2 mRNA levels in MCF‐7/Zol cells.

*ABCG2* expression is regulated at both the transcriptional and posttranscriptional levels through a complex network of signaling pathways and transcription factors. At the transcriptional level, estrogen receptor alpha (ERα), hypoxia‐inducible factor 1 (HIF‐1), peroxisome proliferator‐activated receptor gamma (PPARγ), progesterone receptor (PGR), and the aryl hydrocarbon receptor (AHR) have all been identified as key regulators [36]. Additionally, NF‐κB (p50) has been shown to bind directly to a specific site (−27/ −18) within the *ABCG2* promoter, thereby enhancing its transcription. Interestingly, wild‐type p53 indirectly suppresses *ABCG2* transcription by inhibiting NF‐κB binding to this promoter region [37]. At the posttranscriptional level, microRNAs (miRNAs) such as hsa‐miR‐520h [38], miR‐328 [39], and miR‐212 [40] have been shown to negatively regulate BCRP, contributing to the fine‐tuning of its expression.

NF‐κB is a key molecular link between inflammation and cancer, playing a pivotal role in regulating genes involved in cell proliferation, immunity, survival, metastasis, angiogenesis, and invasion. The NF‐κB signaling pathway is one of the central regulators of drug sensitivity, and has been demonstrated to directly upregulate *ABCG2* expression [41]. NF‐κB not only enhances *ABCG2* transcription but also promotes its membrane localization, facilitating the development of multidrug resistance (MDR) in various cancers, including acute myeloid leukemia and non‐small cell lung cancer (NSCLC) [42, 43]. Moreover, NF‐κB activity is associated with increased cancer cell aggressiveness, including enhanced migration, invasion, and survival [44]. Pro‐inflammatory cytokines such as IL‐1β and TNF‐α further amplify this effect by stimulating both NF‐κB signaling, and *ABCG2* expression in certain breast cancer cell lines, suggesting a coordinated response involved in drug resistance [45]. It also contributes to the resistance of malignant cells to apoptosis by upregulating antiapoptotic genes and downregulating p53 [34]. Notably, the *Bcl‐2* promoter also contains NF‐κB binding sites [35], supporting prior findings of elevated Bcl‐2 mRNA levels in MCF‐7/Zol cells.

Epithelial markers, cytokeratin 18, in particular, were found to be reduced in BCRP overexpressing chemotherapy‐resistant breast tumor tissues. EMT induced via NF‑κB/Snail axis was shown to increase BCRP expression along with mitoxantrone resistance in MCF‐7 cells [46]. Meanwhile, our studies so far suggested that EMT has an important role on the MDR phenotype of resistant MCF‐7 sublines [10]. Therefore, we tested whether MCF‐7/Zol cells had EMT. Vimentin and E‐cadherin immunostaining together with RT‐qPCR results of *VIM*, *CDH1*, and *SNAIL* genes demonstrated that epithelial characteristics of MCF‐7/Zol cells remained, and did not harbor EMT markers.

Elimination of the cytotoxic effect in multidrug resistant cells is achieved gradually over time with the involvement of diverse pathways, dependent on cell type, and may cause or be the result of global phenotypic changes. While the survival or apoptotic pathways may be gradually affected in the process of selection of drug‐resistant clones among heterogeneous cell populations, it is expected that the cellular pathways may also change as the cell populations evolve. Here we report to our knowledge for the first time that (1) BCRP levels are elevated with altered intracellular localization in ZA resistant MCF‐7 cells, (2) BCRP pump causes a decrease in the specific substrate accumulation in the ZA resistant cells whereas no change in intercellular substrate accumulation was observed in parental cells by BCRP inhibition, (3) MCF‐7/Zol cells have increased amount of phosphorylated IκB which is associated with increased nuclear translocation of NF‐κB, and (4) BCRP upregulation together with increased Bcl‐2 levels and decreased apoptotic tendency may be associated with increased nuclear NF‐κB in ZA resistant cells. Elucidation of molecular mechanisms of resistance developed against chemotherapeutic agents is important to target critical pathways and proteins to eliminate the resistant clones as well as for determining biomarkers for MDR.

## Ethics Statement

The authors have nothing to report.

## Conflicts of Interest

The authors declare no conflicts of interest.

## Declaration of Generative AI and AI‐Assisted Technologies in the Writing Process

During the preparation of this study the authors used DeepL Write to improve language and readability. After using this tool/service, the authors reviewed and edited the content as needed and take full responsibility for the content of the publication.

## Data Availability

The data that support the findings of this study are available from the corresponding author upon reasonable request.
